# Effectiveness of Vitamin D and Alpha-Lipoic Acid in COVID-19 Infection: A Literature Review

**DOI:** 10.7759/cureus.59153

**Published:** 2024-04-27

**Authors:** Martin Nguyen, Samuel Aulick, Christopher Kennedy

**Affiliations:** 1 Clinical Sciences, West Virginia School of Osteopathic Medicine, Lewisburg, USA

**Keywords:** corona virus, 1 25 dihydroxy cholecalciferol, alpha lipoic acid, vitamin-d deficiency, sars-cov-2 (covid-19)

## Abstract

Over three years since the World Health Organization (WHO) declared COVID-19 a pandemic, it is still a global burden. Vaccines against COVID-19, caused by SARS-CoV-2, are available and effective for preventing disease. However, their protective effects are not 100%. Currently, the U.S. Food and Drug Administration (FDA) has only approved a limited number of inpatient treatments for COVID-19, such as remdesivir, baricitinib, and tocilizumab. These medications have indications and contraindications applicable to a select patient population. Finding additional effective therapies that are widely available with limited risk could be vital in optimizing treatment strategies for this viral illness. Some vitamins and supplements have been identified as potential options for managing COVID-19. Vitamin D (VD) deficiency has been associated with respiratory tract infections. Moreover, alpha-lipoic acid (ALA) is a powerful antioxidant and helps reduce inflammatory responses in many pathologic conditions. This review aims to analyze the current evidence regarding the effectiveness of VD and alpha-lipoic acid in COVID-19 infection in both outpatient and hospitalized patients.

Relevant randomized controlled trials (RCTs) were identified via the PubMed database from January 1, 2021, to December 31, 2023. Inclusion criteria were as follows: the study design was a randomized controlled trial (RCT), the usage of a constant dose during the intervention period without any additional boluses, and a research ethics committee approved it. Exclusion criteria included a lack of an outcome or apparent intervention, additional boluses, or a single-dose regimen in all the interventional groups. There were 11 studies with a total sample size of 35,717 patients that met the criteria for this review.

A total of 10 RCTs examined the efficacy of VD, and one RCT that reviewed the efficacy of ALA was identified. All of the articles investigated the use of VD or ALA during the treatment of COVID-19. The endpoints of each study varied, including length of stay in hospital, viral load, SARS-CoV-2 infection rate, mechanical ventilation, inflammatory markers, clinical symptoms, Sequential Organ Failure Assessment (SOFA) score, and mortality. In 8/10 VD supplementation trials, significant differences were identified between the interventional and placebo groups in the aforementioned parameters. In 2/10 VD supplementation trials, no significant differences were identified. The ALA supplementation RCT found no differences between the interventional and placebo groups in the SOFA score and 30-day all-cause mortality rate.

The current literature suggests that VD can potentially reduce the SARS-CoV-2 infection rate, oxygen requirements, inflammatory markers, clinical symptoms, and mortality. Regarding ALA, although there was a suggestion of benefit, it was not statistically significant. Common limitations among the different studies included relatively small sample sizes, different geographical patient locations among studies, and differences in dosages. Trials investigating the effects of higher doses of VD supplementation on SARS-CoV-2 infection should be conducted. More research is needed to define best practices and optimal dosing protocols for the use of VD in COVID-19.

## Introduction and background

The severe acute respiratory syndrome coronavirus 2 (SARS-CoV-2), the virus causing COVID-19, is still a major health problem globally. This virus was first detected in China and then reported to the World Health Organization (WHO) in December 2019. Afterward, it was declared a global pandemic by the WHO in March 2020. At the time of writing, it has infected more than 773 million people and has been associated with almost 7 million deaths worldwide [1]. Currently, the U.S. Food and Drug Administration (FDA) has only approved a few treatments including Paxlovid (nirmatrelvir tablets and ritonavir tablets packaged together), intravenous remdesivir, baricitinib, and tocilizumab [2]. However, these therapies only apply to certain populations with a strict selection process. Moreover, under certain circumstances, the FDA may approve the use of unapproved drugs under Emergency Use Authorization (EUA). These drugs can be broadly divided into two groups: antivirals and immune modulators [2].

Alpha-lipoic acid (ALA) is a powerful antioxidant with immunomodulatory activities. ALA regulates several cellular metabolic processes such as adenosine triphosphate (ATP) production and nucleic acid synthesis [3]. Additionally, ALA has been found to possess promising antiviral properties, which has led to interest in exploring its potential use as a therapeutic agent for viral infections, including COVID-19. Vitamin D (VD) is an immunomodulatory hormone with well-established evidence regarding its efficacy in the treatment of various respiratory infections. The benefits of VD stem from its modulation of the immune system, specifically through the suppression of the adaptive immune responses in respiratory epithelial cells during viral infections [4]. Furthermore, VD causes a shift from Th1 cells to Th2 cells, thus decreasing the levels of pro-inflammatory cytokines and limiting immune-mediated injury [4,5]. Thus, there are reasons to explore further use of VD in COVID-19 infections. 

In this review, we examine the state of the current evidence for the use of ALA and VD in the treatment of COVID-19.

## Review

Methods

A literature search was performed on PubMed using the keywords "coronavirus"/"COVID-19"/"SARS-CoV-2" and "vitamin D"/"alpha lipoic acid". The search was refined to include only "randomized controlled trials" (RCT) with a specified date range from January 1, 2021, to December 31, 2023. Inclusion criteria included: the study design as an RCT, the interventional regimen to use a constant dose during treatment without any additional boluses, and the articles must have been approved by a research ethics committee. Exclusion criteria included: lacking outcomes or a clear intervention, using additional boluses or single-dose supplementation in all the interventional groups.

In addition to the identified studies, we incorporated a China-based study by Zhong et al. into our review [6]. This particular study was the only RCT that investigated the clinical efficacy of ALA, resulting in a total of eleven studies included in our review.

Results

Among the 10 RCTs evaluating the efficacy of VD supplementation for COVID-19 infection, 8/10 reported significant differences between intervention and placebo groups.

In one of the first RCTs investigating the effects of VD in the prevention of COVID-19 infection, Villasis-Keever et al. demonstrated that VD supplementation lowered the infection rate of SARS-CoV-2 compared to the control group (6.4% vs. 24.5%, p < 0.001) [7]. A total of 321 subjects were divided into two groups: (1) Supplementation with 4,000 IU VD every day for one month, and (2) using a placebo every day for one month. Interestingly, VD intake provided benefits even though adequate serum VD levels were not reached. However, the dose of VD to optimize the protective effect remained elusive [7].

Additionally, adverse effects (AEs) were comparable between the two groups [7]. Another study by Sabico et al. investigated the effects of different dosages of VD supplementation [8]. Sixty-nine patients hospitalized for mild-moderate COVID-19 infection were randomized to either receive VD with a dosage of 5,000 IU per day or 1,000 IU per day for two weeks [8]. Primary endpoints included fasting blood glucose (FBG), lipids, serum 25(OH)D, and inflammatory markers. The group receiving 5,000 IU showed a significantly shorter time to recovery compared with the 1,000 IU one (6.2 ± 0.8 vs. 9.1 ± 0.8; p = 0.039). Both groups had a significant reduction of IL-6 levels post-intervention. No significant differences in lipid and glucose levels were detected.

In 2022, Sarhan et al. conducted an RCT in Egypt to compare the effects of high-dose VD to low-dose VD in moderate-to-severe COVID-19 patients [9]. A total of 116 patients were divided into two groups: group 1 was given 1 mcg of the VD analog alfacalcidol per day orally (400 IU), and group 2 was given a single dose of 200,000 IU vitamin D3 intramuscularly (IM). All of the standard care for COVID-19 was applied for both groups. The primary outcome was clinical improvement, defined as an improvement in oxygen parameters. Secondary outcomes included hospital stay length, mortality, levels of inflammatory markers, and secondary infections. It was found that the incidence of mechanical ventilation, intensive care unit (ICU) admission, death, sepsis, and atrial fibrillation were significantly reduced in the high-dose group. A significant reduction in inflammatory markers, including CRP, lactate dehydrogenase (LDH), D-dimer, and ferritin, was seen in the high-dose group compared to the low-dose group. Moreover, the high-dose group had a shorter amount of time in hospital (6.1 ± 3.4 vs. 8.9 ± 5.1; p = 0.04) [9].

In a randomized controlled trial with 155 patients requiring respiratory support, Domazet Bugarin et al. found no differences between the interventional group (10,000 IU per day for at least 14 days) versus the control group [10]. In another Norway-based RCT with 34,601 adults, Brunvoll et al. examined the effects of daily low-dose VD (400 IU) supplementation over six months [11]. Primary endpoints included positive SARS-CoV-2 test, serious COVID-19 symptoms (defined as self-reported dyspnea, hospital admission, or death), negative SARS-CoV-2 test, and self-reported acute respiratory infections (ARIs). No differences were identified between the interventional and control groups.

Other trials are summarized in Table 1.

**Table 1:** Summary of primary endpoints and main outcomes of relevant randomized controlled trials. ALA, alpha-lipoic acid; ALT, alanine aminotransferase; ARI, acute respiratory infection; AST, aspartate aminotransferase; CRP: C-reactive protein; ICU, intensive care unit; LDH, lactate dehydrogenase; LOS, length of hospital stay; NLR, neutrophil-to-lymphocyte ratio; RCT, randomized controlled trial; SOFA score, Sequential Organ Failure Estimate score; VD, vitamin D.

	Study design, dosage	Sample size and notable features	Primary endpoints	Main results (intervention vs. placebo)	Conclusion	
Supporting the use of vitamin D						
Villasis-Keever et al. (2022) [7]	Double-blind RCT, 4000 IU daily for 30 days	N = 321; healthcare workers from 4 hospitals in Mexico City	SARS-CoV-2 infection rate	6.4% vs. 24.5% (p = 0.008)	VD supplementation is effective in prevention of SARS-CoV-2 infection in highly exposed individuals regardless of VD status	
Sabico et al. (2021) [8]	RCT; 2 groups: (1) 5,000 IU/d , (2) 1,000 IU/d (for 2 weeks)	N = 69; hospitalized for mild-moderate COVID-19	Fasting blood glucose, lipids, serum 25(OH)D, and inflammatory markers	-Group 1 has a significantly shorter time to recover than group 2: 6.2 days vs. 9.1 days (p < 0.05) -Decrease in IL-6 in both groups	The authors recommended vitamin D3 (5,000 IU/d) as an adjuvant therapy in patients with suboptimal VD status	
Sarhan et al. (2022) [9]	Prospective RCT. Oral vitamin D3 was administered to group 1 (1 mcg/day) and intramuscularly (200,000 IU) to group 2	N = 116	-Improvement in oxygen parameters -Secondary outcomes: hospital stay length, mortality, variation in inflammatory markers, CRP, LDH, ferritin, D-dimer, secondary infections, or at least one adverse event	-Incidence of mechanical ventilation, ICU hospitalization, death, sepsis, and atrial fibrillation was significantly reduced in group 2 -CRP, LDH, D-dimer, ferritin, AST/ALT were significantly reduced in group 2 -Significantly shorter amount of time spent in hospital in group 2	High-dose vitamin D3 was associated with better clinical outcomes and fewer adverse outcomes compared to those receiving low-dose treatment	
Bishop et al. (2022) [12]	Double-blinded RCT, utilizing extended released calcifediol (ERC) for 4 weeks. Day 1-3: 300 mcg/d; day 4-30: 60 mcg/day	N = 160	-Serum 25(OH) levels (≥ 50 ng/mL) -Secondary endpoints: resolution time, respiratory symptoms	-81% vs. 15% (p < 0.0001) that has target serum 25(OH)D -Respiratory symptoms were resolved 4d faster in the intervention group	ERC safely raised serum VD ≥ 50 ng/mL in outpatients with mild-moderate COVID-19 and possibly shortened recovery time of respiratory symptoms	
Torres et al. (2022) [13]	Multi-center, single-blind, prospective RCT -Group 1: 10,000 IU/day of cholecalciferol for 14 days -Group 2: which received 2,000 IU/day for 14 days.	N = 82	-Increase of 25(OH)D serum level ≥ 30 ng/mL (after 14 days) -Secondary endpoints: -inflammatory profile -LOS -Cytotoxic response	-39.02% vs. 9.09% (p = 0.0027) that get adequate serum 25(OH)D -LOS: 8 days vs. 29.2 days (p = 0.0381) -IL-10 (anti-inflammation) and IFNγ (antiviral) significantly increased in group1 (vs. group 2) -CD4+ cells is significantly higher in group 1 (vs. group 2) -Cytotoxic response was significantly higher in group 1 (4 times higher than group 2)	Administration of 10,000 IU cholecalciferol as an adjuvant of standard care during hospitalization of COVID-19 patients may improve the inflammatory profile, cytotoxic responses, LOS, and possibly improve prognosis	
Rastogi et al. (2022) [14]	RCT. Group 1: 60,000 IU/d of cholecalciferol for 7 days Group 2: placebo	N = 40	Proportion of patients with SARS-CoV-2 RNA negative before day 21	-SARS-CoV-2 RNA negative: 62.5% vs. 20.8% (p = 0.018) -Significant reduction in fibrinogen (p < 0.01)	Administration of high doses of cholecalciferol significantly reduced the viral load in those infected with SARS-CoV-2 as well as their fibrinogen levels	
Elamir et al. (2022) [15]	Open-label RCT. Intervention group: 0.5 mcg/day for 14 days and were compared to those receiving no intervention	N = 50	-Oxygen requirements, LOS, ICU admission, mortality, and readmission	-Change in O2 saturation SaO_2_/FIO_2_ ratio: group 1 +91.04 (± 119.08) vs. group 2 +13.2 (± 127.7) (p = 0.03). This demonstrates a significant clinical improvement -No statistical significance regarding other outcomes	The sample data was not large enough to draw significant conclusions, but the available data suggests that calcitriol may improve oxygenation among hospitalized patients with COVID-19	
						Maghbooli et al. (2021) [16]	Multicenter, double-blinded, RCT. 2 groups: (1) standard care + 3,000-6,000 IUs/d of vitamin D3; (2) standard care + placebo	N = 106	-Circulating VD levels (>30 ng/mL) -Mortality -ICU duration -Inflammatory response -Ventilator assistance	-30 day: 79.4% vs. 12.5% (p < 0.001) with VD levels > 30 ng/mL -60 day: 100% vs. 10.5% (p < 0.001) -ICU duration, mortality, and ventilation: not significant -Intervention group had a significant increase in lymphocyte percentage and lower NLR -Lower NLR was associated with reduced ICU admission and mortality -No adverse reactions in Tx group	The administration of 25(OH)D3 proved to be clinically significant in improving immune function by increasing blood lymphocyte percentage	
Not supporting the use of vitamin D						
Domazet Bugarin et al. (2023) [10]	Single center, open-label RCT. Patients in the intervention received 10,000 IU of cholecalciferol for at least 14 days	N = 155	Number of days spent on respiratory support (invasive or non-invasive)	No significant difference between the two groups	VD supplementation in patients admitted to the ICU for COVID-19 infections did not reduce the number of days that respiratory support was required	
Brunvoll et al. (2022) [11]	Quadruple-blinded, randomized placebo-controlled trial. Intervention: cod liver oil (400 IU of VD/day vs. placebo for 6 months)	N = 34,601	Positive SARS-CoV-2 test; serious COVID-19 (dyspnea, hospital admission, death); negative SARS-CoV-2 test; self-reported ARIs	No significant difference between the two groups	Supplementation did not reduce the likelihood of contracting SARS-CoV-2	
						Effects of ALA						
Zhong et al. (2022) [6]	A randomized, single-blind, group sequential, active-controlled trial Group 1: 1,200mg/day ALA (IV) once plus standard care for 7 days Group 2: saline plus standard care for 7 days.	N = 17; critical cases of COVID-19 infection	-SOFA scores Secondary endpoints: -30-day all-cause mortality	At 7 days: SOFA = 4 vs. 6 (p = 0.36) 30-day all-cause mortality: 37.5% vs. 77.8% (p = 0.09)	ALA is associated with lower SOFA score and lower 30-day all-cause mortality (vs. placebo). However, due to the small sample size, there was no statistical significance	

Discussion

Epidemiology of Vitamin D Deficiency

Hydroxyvitamin D (25(OH)D) represents the total concentration of both 25-hydroxyvitamin D2 and 25-hydroxyvitamin D3 [17].

In 1998, Malabanan et al. defined VD deficiency as a blood level of 25(OH)D being less than 20 ng/mL [18]. More recently, in practice guidelines of The Endocrine Society, VD deficiency was defined as a 25(OH)D < 20 ng/mL, insufficiency as 21-29 ng/mL, and sufficiency as ≥ 30 ng/mL for maximal musculoskeletal health [19]. This definition has gained some popularity and currently has been accepted by the American Geriatric Society, American Association for Clinical Endocrinologists, and National Osteoporosis Foundation [20,21].

VD deficiency is now considered a global health problem [17,22,23]. In the U.S., it is estimated that 36% of the general population has VD deficiency, and among pregnant women, VD deficiency prevalence is 27-91% [17]. Among middle-aged and older adults in the U.S., there is approximately 20% prevalence of VD deficiency with a serum level < 50 ng/mL [24]. VD deficiency has also been shown to be associated with increased mortality risk [24]. Populations with increased risk of VD deficiency included pregnant women, people of color (e.g. African Americans, Hispanics), obese patients, people with little sun exposure, and those who are physically inactive [22,23]. In a prospective study including 40 healthy pregnant women in Boston, even with VD supplements throughout the pregnancy (average 600 international units (IUs) daily), Lee et al. reported 50% of mothers and 65% of their newborn infants as VD deficient with a blood level < 20 ng/mL [25]. Thus, a dosage of 600 IUs of VD daily may not sustain serum 25(OH)D > 20 ng/mL for this particular group.

Vitamin D and Respiratory Infections (Including COVID-19)

In a Finnish study, Laaksi et al. investigated a possible association between VD deficiency (< 16 ng/mL) and acute respiratory tract infections (ARTIs) in 800 young men serving on a military base [26]. It was shown that subjects with 25(OH)D concentrations < 16 ng/mL had a 63% increased risk of absence from duty due to ARTIs compared to individuals with serum 25(OH)D ≥ 16 ng/mL (p = 0.004). Based on the results of this study, the same group of researchers conducted a randomized controlled trial (RCT) in which the subjects either received 400 IU of vitamin or placebo for six months (from October to March). The results showed that the number of men remaining healthy was significantly higher in the intervention group than in the control group (51.3% vs. 35.7%, p = 0.045) [27]. In 2013, Bergman et al. conducted a meta-analysis including 11 RCTs with 5560 patients [28]. It was shown that VD had a protective effect against respiratory tract infections (RTIs) (odds ratio (OR), 0.64; 95% CI 0.49-0.84), and the protection is more significant in studies utilizing once-daily dosing compared to bolus doses (OR = 0.51 vs OR = 0.86, p = 0.01). In simple terms, daily dosing regimens showed a 3.5 times greater reduction in OR of RTIs than bolus approaches. The authors concluded that VD could be an effective way to prevent RTIs, and one of the key factors appeared to be dosing interval (daily dosing vs. bolus). Therefore, this may help explain why many studies using bolus regimens of VD failed to show any significant differences between intervention versus controlled groups [28]. In a probability survey of the US population that included almost 19,000 participants aged 12 or older, Ginde et al. found that there was an inverse association between serum 25(OH)D and recent upper respiratory tract infections (URTIs) [29]. Moreover, the authors suggested that the role of VD may be even more important in individuals with respiratory tract diseases such as asthma and chronic obstructive pulmonary disease (COPD) due to an increased risk of RTIs in these patients [29-31]. In a meta-analysis including 25 high-quality RCTs with 113,121 patients, Martineau et al. concluded that VD supplementation has significant protective effects against overall ARTIs [32]. Those who benefited the most from VD supplementation were the individuals with serum 25(OH)D < 10 ng/mL and those receiving VD daily or weekly without any additional boluses [32]. Those results are consistent with another meta-analysis by Bergman et al., and that was the reason for focusing on those RCTs with constant doses during the treatment period without any additional bolus in this review [28].

The above results on RTIs suggest at least two considerations regarding VD supplementation. Firstly, VD plays a clinically important role in RTIs and has considerable protective effects against RTIs [26-29]. These effects will undoubtedly drive interest in research communities in the near future in addition to the well-established effects of VD on the musculoskeletal system. Secondly, attention regarding VD supplementation should be exercised in healthy adults and children, not just in infants and the elderly.

Regarding COVID-19 infections, most cases are mild and resolved without complication. The infection-hospitalization ratio (IHR), defined as the percentage of infected individuals who are hospitalized, was estimated to be 2.1% [33]. Severe cases are associated with acute respiratory distress syndrome (ARDS), sepsis, and multiorgan failure. Furthermore, 71% of severe cases require mechanical ventilation [34]. The predominant focus has been on the development of effective vaccines. However, it is also necessary to identify therapeutic options that may reduce the risk of contracting the disease or reduce the severity of symptoms [35,36]. The overlap between risk factors for severe COVID-19 disease and VD deficiency including obesity, old age, and Black or Asian origin, has led to a postulation that VD supplementation could play an important role as a preventive or therapeutic option for COVID-19 [35]. VD is known for its ability to enhance the innate immune system by inducing antimicrobial peptides (AMPs) such as cathelicidin which increases viral destruction, thus making infection of SARS-CoV-2 and development of symptoms less likely. Moreover, VD may help reduce the inflammatory response to SARS-CoV-2 [37].

Since the outbreak of the COVID-19 pandemic, there have been multiple studies that investigated the association between VD and COVID-19. Ilie et al. reported an association between VD deficiency and SARS-CoV-2 infection as well as COVID-19 mortality [38]. The authors suggested VD supplementation may improve the overall clinical outcomes and protect against SARS-CoV-2 infection. Subsequently, Mok et al. have supported that postulation by reporting the active form of VD (1,25-dihydroxy vitamin D) has a strong inhibitory effect against SARS-CoV-2 in human nasal epithelial cells [39]. They also demonstrated the addition of VD helps cells upregulate the genes important for inhibiting viral replication, such as 24(OH)ase and LL-37 [39]. In another study comprised of 235 inpatients, Maghbooli et al. found that among the cases that succumbed to COVID-19, 9.7% had a sufficient level of VD (≥ 30 ng/mL) (p=0.04) [40]. Moreover, only 6.3% of all mortality cases have a 25(OH)D concentration of at least 40 ng/mL. Additionally, serum CRP was lower, and the lymphocyte percentage was higher in patients with sufficient VD [40]. In a pilot RCT by Entrenas Castillo et al. high-dose calcifediol, a main metabolite of VD, significantly reduced the rate of ICU admission of hospitalized patients due to COVID-19 [41]. These findings suggest that adequate VD levels have the potential to improve immune system function and that VD supplementation may have the potential as an adjuvant therapy for patients with COVID-19 infections.

Regarding the risk of COVID-19 infection and VD levels, in a study of 7,087 Israel patients, Merzon et al. reported there was an association between low plasma of 25(OH)D (< 30 ng/mL) and increased likelihood of COVID-19 infection (OR 1.58, 95% CI 1.24-2.01, p < 0.001) [42]. Moreover, they concluded low plasma VD levels appeared to be an independent risk factor for COVID-19 infection and hospitalization, regardless of demographic characteristics and past medical conditions. In one of the largest observation studies with over 190,000 patients, Kaufman et al. reported that SARS-CoV-2 infection is strongly and inversely associated with circulating levels of 25(OH)D regardless of geography, age, sex, and ethnicity [43].

In this review, we found two studies with no significant difference between the interventional group versus controlled group. However, Domazet Bugarin et al. acknowledged that one of the main limitations of that study was the small sample size [10]. Initially, they wanted to recruit at least 274 patients to be able to detect a two-day difference in the primary outcome. Therefore, they suggest the available results would serve for larger and more powerful studies in the future [10]. The other study by Brunvoll et al. also reported that there was no difference when using 400 IUs of VD daily for six months [11]. However, we think there may be several reasons that could help explain this result. First, as mentioned above, 600 IUs daily is not adequate to maintain a serum level of 25(OH)D above 20 ng/mL. Second, to achieve a 25(OH)D level of 40-50 ng/mL, an average adult needs to ingest 4,000-5,000 IUs of VD daily [44,45]. Moreover, The Endocrine Society suggested that 4,000 IUs daily is reasonable [19]. Third, Sarhan et al. have demonstrated that high-dose VD supplementation is more effective than low-dose supplementation (1 mcg alfacalcidol/day, similar to 400 IUs/day) [9]. Although an optimal dose of VD still is a topic for debate, 400 IUs daily may be insufficient, even given the long period of intervention (six months) in that trial [11].

Treatment and Prevention of Vitamin D Deficiency

The target level is to obtain a serum 25(OH)D of at least 30 ng/mL. VD toxicity is extremely rare, and there are no reported cases of VD intoxication with serum levels < 200 ng/mL or with oral input < 30,000 IU/d [17,20]. The safety of VD supplementation has been proven in a meta-analysis conducted by Martineau et al. [32]. Daily or weekly regimens seem to be more beneficial than bolus regimens [28,32]. The Endocrine Society suggested that 4,000 IUs daily should be the upper limit for children and 10,000 IUs daily the upper limit for adults [19]. However, for obese patients, they may require 2-3 times more VD than usual to prevent VD deficiency [19].

Alpha-Lipoic Acid and COVID-19 Infection

ALA is a natural compound that can be found in virtually all animal species. Moreover, it has been shown that ALA is a natural and very powerful free radical scavenger [46]. In addition to its antioxidant effects, ALA helps with the regeneration of glutathione (GSH), vitamin C, vitamin E, and coenzyme Q10 in vivo [47]. Additionally, ALA and its metabolites increase GSH synthesis and support recovery from oxidative damage [47]. The tolerability and safety of oral supplementation have been confirmed [48,49]. ALA has also been shown to reduce systemic inflammatory response in acute coronary syndromes and liver transplantation [50,51].

At the time of writing, there is only one RCT conducted in Wuhan, China that investigated the effects of ALA in COVID-19 patients. Zhong et al. recruited 17 critical cases of COVID-19 in a hospital, then randomized and divided them into two groups: (1) standard care + ALA (1200 mg/d, intravenous infusion) for seven days, and (2) standard care + normal saline for seven days [6]. The primary outcome was the Sequential Organ Failure Estimate (SOFA) score, and the secondary outcome was all-cause mortality at 30 days. The results showed that SOFA score was 4.00 ± 2.24 vs. 6.00 ± 3.35, respectively (p = 0.36). All-cause mortality was 37.5% in the ALA group vs. 77.8% in the placebo group (p = 0.09). The authors noticed that there was a clear tendency of improvement in the intervention group, but due to limited sample size, it could not reach statistical significance. Thus, they suggested that further studies with larger sample sizes should be designed to investigate the role of ALA in severe cases of COVID-19 [6].

Limitations

Regarding VD supplementation, it is challenging to assess the effects of VD in severe COVID-19 cases. First, when patients with severe disease, such as ICU patients, it may be already too late for them to receive any benefits of VD supplementation [35]. Second, corticosteroids, such as dexamethasone, are now considered first-line therapy in many severe diseases. Thus, the benefits of VD supplementation could be masked in patients on concomitant corticosteroid therapy. Third, VD may prove to be best applied in population-based trials where the authors could investigate its benefits in preventing or decreasing the severity of COVID-19 before the patients are infected with the virus. Additionally, because our review comprised many trials from various locations, it is possible that different strains of COVID-19 are being compared across studies. Fourth, this review was only conducted based on one database (PubMed). Therefore, it may be improved if we could have combined it with other databases such as Web of Sciences or Scopus.

## Conclusions

Based on the current literature, we do not recommend vitamin D (VD) supplementation in the routine treatment of COVID-19 infection as a standalone therapy. At best, VD status may be one of the many factors which could affect the outcome of COVID-19. However, it is a problem that could be addressed safely at a low cost. There is minimal risk in improving the population's VD status. However, it is important to detect the reasons why the patients are VD deficient. Potential causes include they were not taking enough VD from food or sunlight exposure. Thus, we may tailor an individualized approach for each patient. Additionally, there is almost no harm from VD supplementation, and even marginal benefits could be targeted with inherently minimal risk. Further studies investigating the combined effects of both VD and alpha-lipoic acid for COVID-19 patients could provide information on this approach. Future studies evaluating the effects of different doses of VD supplementation (e.g., 1000 IUs/ day, 2000 IUs/day, or more in adults) would also be beneficial.
